# Disparities in a provision of in-hospital post-arrest interventions for out-of-hospital cardiac arrest (OHCA) in the elderly population—protocol for a systematic review

**DOI:** 10.1186/s13643-016-0234-4

**Published:** 2016-04-07

**Authors:** Joanna M. Bielecki, Josephine Wong, Nicholas Mitsakakis, Prakesh S. Shah, Murray D. Krahn, Valeria E. Rac

**Affiliations:** Leslie Dan Faculty of Pharmacy, University of Toronto, Toronto, ON Canada; Toronto Health Economics and Technology Assessment (THETA) Collaborative, Toronto General Research Institute (TGRI), University Health Network, Toronto General Hospital, Eaton Bldg., 10th Floor, 200 Elizabeth Street, M5G 2C4 Toronto, ON Canada; Department of Pediatrics, Mount Sinai Hospital, 600 University Avenue, M5G 1X5 Toronto, ON Canada; Department of Pediatrics, University of Toronto, Toronto, ON Canada; Institute of Health Policy, Management and Evaluation, University of Toronto, Toronto, ON Canada

**Keywords:** Out-of-hospital cardiac arrest, OHCA, Post-resuscitation interventions, Elderly, Disparity

## Abstract

**Background:**

Out-of-hospital cardiac arrest (OHCA) is a significant cause of death in developed countries. The majority of OHCA patients are elderly (≥65 years), and it was documented that they were less likely than younger patients to receive the evidence-based interventions, even though the improvement in survival in the elderly age group was higher than in younger population. Our goal is to investigate any disparity in the provision of post-arrest care for the elderly with OHCA and a sustained return of spontaneous circulation (ROSC).

**Methods/design:**

Eight relevant, electronic databases will be systematically searched to identify eligible studies. The searches will be supplemented with gray literature searching of theses, dissertations, and hand searching of pertinent journals. Two independent reviewers will screen the titles and abstracts and select studies for full text analysis using Preferred Reporting Items for Systematic Reviews and Meta-analyses (PRISMA) method, and both will extract information from the selected studies employing a form based on the Data Extraction Template for Cochrane Reviews. A team of three reviewers will assess the quality of the studies with the modified Downs and Black scale. Statistical methods for evidence synthesis, such as meta-analysis and meta-regression, will be applied to compare and combine the evidence regarding the association between age and intervention provision/utilization, adjusting for a number of significant confounders, such as patient characteristics and co-morbidities and availability of intervention techniques, as well as study specific characteristics. The strength of evidence from the selected studies will be assessed using a modified Grades of Recommendation, Assessment, Development, and Evaluation (GRADE) system.

**Discussion:**

The findings obtained from this systematic review should inform whether disparity exists in the provision of post-arrest care for the elderly (≥ 65 years old) with OHCA or not. Addressing this problem has a potential to substantially increase the number of > 65-year-old, long-term survivors. The results of our review might also point to the gaps in the published literature that specifically examines disparity in provision of care for this population. This systematic review was designed in accordance with the Preferred Reporting Guidelines for Systematic reviews and Meta-analyses (PRISMA statement), while the protocol follows the Preferred Reporting items for Systematic review and Meta-analysis protocols (PRISMA-P) statement.

**Systematic review registration:**

PROSPERO CRD42015027822

**Electronic supplementary material:**

The online version of this article (doi:10.1186/s13643-016-0234-4) contains supplementary material, which is available to authorized users.

## Introduction

### Background

Out-of-hospital cardiac arrest (OHCA) is a significant cause of death in developed countries. The reported incidence is highly regionally variable. In the USA, it was estimated to be about 424,000 emergency medical services (EMS) assessed OHCAs per year [1–3]. In 2011, the survival rate to hospital admission was 26.3 % [4] and the survival to hospital discharge was 10.4 % [1]. The estimated global incidence was 55 per 100,000 person-years, with a survival to discharge rate of 7 % [5]. In Ontario, Canada, the overall number of OHCA patients transported alive to hospital was 36 cases per 100,000 persons per year. This rate did not change significantly from 2002 to 2011 [6]. However, both the 30-day survival and the 1-year survival improved significantly from 8.8 to 19.1 % and 7.6 to 18.2 %, respectively, over the same period [6].

The 2015 American Heart Association Guidelines for post-cardiac arrest care recommended emergent coronary angiography be performed for OHCA patients with suspected cardiac etiology of arrest; targeted temperature management (TTM) between 32 and 36 °C for comatose adult patients with return of spontaneous circulation (ROSC); and the earliest time for neuroprognostication to be 72 h after return to normothermia for patients treated with TTM and 72 h after cardiac arrest for patients not treated with TTM [7]. Depending on the cause of cardiac arrest, post-arrest interventions such as coronary artery bypass graft (CABG) [8–11] and placement of implantable cardioverter-defibrillators [12–14] have been found to be associated with better survival.

The majority of OHCA patients are elderly (≥ 65 years). The median age for OHCA was 67 (53–80) in North America [3], and the proportion of OHCA victims ≥ 65 years in the US Cardiac Arrest Registry to Enhanced Survival (CARES) was 51.1 % [4]. There is a wide variability in characteristics and outcomes among OHCA patients [15]. Most studies reported an inverse relationship between survival and age [16–18]. Yet, it was found that increasing age was associated with a significant increase in the chance of being admitted to hospital with the return of spontaneous circulation (ROSC) [16, 18]. In most reports, co-morbid conditions, polypharmacy, do-not-resuscitate (DNR) status, and access to post-resuscitation interventions have not been evaluated and controlled for. A recent study showed that cardiocerebral resuscitation for OHCA was associated with better survival rate and neurological outcomes across age groups, with older patients showing a marked survival benefit with neurologic preservation [19].

As the world population is aging, the incidence of OHCA is expected to rise. The number of people aged 65 or older is projected to triple, from an estimated 524 million in 2010 to nearly 1.5 billion in 2050 [20–22]. There will be an increasing need for health care provisions for the elderly globally. Yet, most models of care are not optimized for the elderly [23]. In the case of acute coronary syndrome, which mostly affects the elderly and is the most common cause of OHCA [24], the outcome is highly correlated with the use of evidence-based interventions [25–29]. Yet, numerous studies have shown that elderly patients were less likely than younger patients to receive evidence-based interventions even though they were eligible and would benefit [28–41]. This might explain the age disparity in outcomes observed in some studies [37, 42].

In view of the increasing evidence that advanced age is not associated with worse outcome for cardiovascular procedures (e.g., early angiography [43], primary percutaneous coronary intervention (PCI) [44], coronary artery bypass graft (CAGB) surgery [45], implantable cardioverter-defibrillator (ICD) placement [46–48], aortic valve replacement [49], anticoagulant therapy [50]), the withholding of these procedures on the basis of risk associated with advanced age is no longer justifiable [51]. In the case of acute coronary syndrome, an early invasive strategy with revascularization was associated with improvement in survival and outcomes for all age groups, with greater absolute accrued benefit in the elderly [39]. The variations in outcomes in the elderly population are more likely to be related to the entire care process (intraoperative and postoperative management), rather than specific procedural issues and preoperative co-morbidities [52–54].

Since the elderly are associated with a greater chance of being admitted to hospital with ROSC with a lower survival to discharge rate [16], this has led us to question whether an underutilization of evidence-based interventions for post-resuscitative care exist for this population. In an Ontario population-based cohort study of 34,291 OHCA patients transported alive to an acute hospital from 2002 to 2012, Wong, et al. [6] found discrepancies in the improvement in survival by age group. For younger patients (age 20–49), the unadjusted 30-day and 1-year survival improved from 9.2 to 18.7 % and 8.1 to 17.7 %, respectively. Whereas, for patients age 65–74, the unadjusted 30-day and 1-year survival only improved from 10.4 to 13.1 % and 9.0 to 11.2 %, respectively. Whether this discrepancy was due to differences in treatment aggressiveness and/or effectiveness remains to be investigated.

Irrespective of age, long-term survivors can expect a good quality of life after early and successful resuscitation comparable to the general population [55–60] and those > 80 years old show a marked survival benefit with neurologic preservation [19]. These findings have led us to question the appropriateness of applying DNR status in the event of OHCA.

### Objectives

The objective of this systematic review is to investigate any disparity in the provision of post-arrest care for the elderly (≥ 65 years old) with OHCA and ROSC after hospital admission.

## Methods

The systematic review will be designed in accordance with the Preferred Reporting Items for Systematic reviews and Meta-analyses (PRISMA statement) [61], while the protocol followed the PRISMA-P statement and utilized the PRISMA-P checklist (Additional file 1) [62, 63]. This protocol is registered with PROSPERO as CRD42015027822.

### Eligibility criteria

#### Study designs

This systematic review will include randomized controlled studies (RCTs), prospective and retrospective cohort studies, case-control, case series and cross-sectional studies, and published, peer-reviewed registry data of OHCA patients who survived to hospital admission. The review will include studies analyzing populations stratified by age. If a study is based on a single cohort group without age stratification, the study will be excluded, unless the authors of the study can provide either age-stratified analyzed or the raw, anonymized data associated with age parameter. If in the selected studies analyzed interventions are combined, we will contact authors and ask for separate intervention-based analysis or raw, anonymized data to analyze ourselves.

#### Interventions

Based on the 2015 and 2010 AHA Guidelines for post-cardiac arrest care [7, 64, 65] and 2008 AHA Consensus Statement on post-cardiac arrest syndrome [24], the focus shall be on the provision of the following key evidence-based interventions: therapeutic hypothermia or TTM [66–71], emergent coronary angiography and percutaneous coronary intervention (PCI) [72–74], and neuroprognostication ≥ 72 h [65, 75, 76]. In eligible patients, interventions such as coronary artery bypass graft (CABG) [8–11] and placement of implantable cardioverter-defibrillators [12–14] will also be considered.

#### Participants

The population will consist of patients who were admitted to hospital after suffering an OHCA and experienced ROSC. The review will focus specifically on the differences in utilization rates of these procedures with respect to age (≥ 65 and < 65 age groups). If data on do-not-resuscitate (DNR) status are available, patients will be excluded from the final analysis.

#### Outcomes

The outcomes of interest are the rates of utilization of these evidence-based interventions.

#### Comparators

Our comparator group constitutes of a population < 65 of age, with OHCA who survived hospital admission.

#### Setting

Restrictions on the type of setting will not be imposed.

#### Language

Only English language publications will be considered. However, a selection of possibly eligible articles in other languages will be included in the appendix.

### Information sources

Eligible studies will be identified through a systematic comprehensive search of the following databases: MEDLINE (Ovid), EMBASE (Ovid), CINHAL (Ebsco), Cochrane Database of Systematic Reviews (CDSR), DARE (Database of Abstracts of Reviews of Effectiveness), LILACS, International Clinical Trials Registry Platform (ICTRP), Science Citation Index Expanded (SCI-EXPANDED), and Conference Proceedings Citation Index-Science (CPCI-S), as well as Dissertations & Theses at University of Toronto and ProQuest Dissertations & Theses Global. Since some interventions started to be used in the 1990s, a database search from 1989 onward is considered sufficient. PROSPERO repository will also be searched for all active or completed systematic reviews. Electronic database searches will be supplemented with consultations with authors of unpublished studies, inspection of reference lists of relevant articles, and hand searching of pertinent journals.

### Search strategy

The search strategy was designed and conducted by an information specialist experienced in systematic reviews (JB) following the Cochrane systematic review methodology [77]. It includes controlled vocabulary (MeSH) and natural language terms in the following concept areas: heart arrest; eligible interventions; health services accessibility, provision, and disparity; elderly; and synonyms. The final search strategy will be peer reviewed by an independent medical information specialist (Melanie Anderson), using Peer Review of Electronic Search Strategies (PRESS) checklist [78]. The search will be updated closer to the end of our review to ensure that the most recent eligible articles are captured. A detailed search strategy for MEDLINE (Ovid) is provided in the Table 1. The final Medline strategy will be translated into syntax appropriate for each database used.

**Table 1:** Medline Ovid proposed search strategy

1.	*heart arrest/or *out-of-hospital cardiac arrest/or (asystole? or ((heart or cardiac or post-cardiac or cardiopulmonary or cardio-pulmonary) adj2 arrest) or post-arrest or ((cardiac or heart) adj3 (sudden or out-of-hospital or "out of hospital") adj3 arrest) or OHCA).tw,kf.
2.	exp cardiac catheterization/or exp angioplasty balloon/or angioplasty, laser/or exp hypothermia, induced/or exp defibrillators, implantable/or exp myocardial revascularization/or transmyocardial laser revascularization/or circulatory arrest, deep hypothermia induced/or angiocardiography/or cardiac catheterization/or cardiography, impedance/or coronary angiography/or exp percutaneous coronary intervention/or exp coronary artery bypass/or ((catheter$ adj2 (heart or cardiac)) or ((balloon or transluminal) adj3 angioplast$ adj3 (coronary or laser)) or (induced adj3 hypothermia?) or (defibrillator? adj3 implantable) or ICD or (revasculari#ation? adj2 (myocardial or transmyocardial or laser)) or "internal mammary arter$ implantation" or (("deep hypothermic" or "deep hypothermia") adj3 circulatory adj3 arrest) or (function adj3 test? adj3 (cardiac or heart)) or "chain of survival" or angiocardiograph$ or ((cardiograph$ or transthoracic) adj3 impedance) or (coronary adj angiograph$) or ((technique? or technic? or stud?) adj2 (cardiac or intracardiac) adj2 electrophysiologic$) or (electrogram adj ((bundle adj2 his) or intracardiac or atrial)) or (programmed adj2 electrostimulation adj2 cardiac) or (coronary adj4 (intervention? or revasculari#ation?) adj4 percutaneous) or ((neurological or neurologic) adj prognostication) or neuroprognostication).tw,kf.
3.	health services accessibility/or healthcare disparities/or delivery of health care/or delivery of health care, integrated/or exp rural health/or exp rural health service?/or resuscitation orders/or advance directive adherence/or withholding treatment/or refusal to treat/or refusal to participate/or health facility size/or coronary care units/or medically underserved area/or health services research/or quality of health care/or program evaluation/or exp quality indicators, health care/or utilization review/or concurrent review/or health impact assessment/or "health care quality, access, and evaluation"/or rural health services/or urban health services/or health services for the aged/or exp geriatric nursing/or exp geriatric assessment/or cardiovascular nursing/or critical care nursing/or triage/or intensive care/or critical care/or intensive care units/or ((access$ adj3 (program or "health service?")) or (distribution?? adj3 (activit$ or community-based or "community based")) or (rural adj3 health) or (("do-not-resuscitate" or "do not resuscitate" or resuscitat$) adj3 (order? or decision? or polic$ or withhold$)) or ((adherence? or compliance) adj3 ("advance directive" or directive?)) or ((withdraw$ or cessat$ or withhold$) adj3 (treatment? or care)) or (health adj2 attitude?) or (("physician? refusal" or refusal) adj3 treat) or (size? adj3 ((health adj3 facility) or hospital?)) or (((medical$ adj3 underserved) or (physician? adj3 shortage)) adj3 (area? or region? or location or district? or neighbo?rhood)) or (("health services" or "medical care" or healthcare or "health care") adj3 (research or quality or indicator? or outcome? or stud$ or (process? adj3 (measure? or assessment?)))) or (("health services" or "medical care" or healthcare or "health care") adj3 (inequit? or equit? or access or disparit$ or deliver$)) or ((patient or patient-centered) adj3 outcome? adj3 (assessment? or research)) or (program$ adj3 (evaluation? or effectiveness or sustainabilit$ or appropriateness)) or (review? adj (concurrent or "continued stay" or utili#ation)) or (impact adj2 assessment? adj2 health) or (health adj3 service? adj3 (geriatric or aged or elderly or urban)) or (geriatric adj2 (assessment? or nursing?)) or ((coronary or cardia$ or cardio$ or vascular or intensive or critical) adj3 (nursing or care or unit?)) or (health adj3 care adj3 evaluation?) or triage? or (provision? adj3 care)).tw,kf.
4.	exp aged/or "aged, 80 and over"/or frail elderly/or exp geriatrics/or (geriatric$ or gerontology or elder?? or senior? or retiree$ or retirement? or sexagenarian$ or septuagenarian$ or octogenarian$ or nonagenarian$ or centenarian$ or supercentenarian$ or ((old?? or elder?? or age? or senior? or geriatric?) adj2 (patient? or adult$ or client$ client$ or patient$ or person$ or citizen? or people or population? or frail or functionally-impaired or "functionally impaired")) or grandparent$ or grandmother$ or grandfather$ or grandma?? or grandpa?? or ((old??? or advance?) adj2 (old or age?)) or ((age? or old??) adj2 ("6# years" or "7# years" or "8# years" or "9# years" or "10# years"))).tw,kf.
5.	1 and 2 and 3 and 4
6.	limit 5 to yr = "1989 -Current"
7.	Animals/not (Animals/and Humans/)
8.	6 not 7

### Data management

The results of the literature search will be collected in a separate EndNote library, deduplicated, and screened according to the study selection process. The team will adapt the Data Extraction Template for Cochrane Reviews [79] to create the study eligibility form. The review team will design a coding schema used at successive stages of the review process. The eligible studies will receive appropriate codes within EndNote reference management software to indicate their status after title/abstract and full text screenings.

### Study selection and screening process

Two reviewers (JB and JW) will be following a hierarchical screening method adapted from Preferred Reporting Items for Systematic Reviews and Meta-Analyses (PRISMA) [61]. Both reviewers will independently screen the titles and abstracts of the studies identified by the search strategy and select studies for full text analysis, based on study design and study population. The full text from initially selected studies will be analyzed by the same reviewers according to the above stated eligibility criteria. Whenever necessary, the study authors will be contacted to inform our decision about study eligibility. Studies will be excluded from selection if they are based on unpublished and non-peer-reviewed registry data or if the publication is an abstract, commentary, editorial, or a letter to the editor. Both reviewers will keep records of the reasons for exclusion in a data extraction form based on the Data Extraction Template for Cochrane Reviews [79]. Neither author will be blinded to the journal titles or to the study authors or institutions. Any disagreements at any stage will be recorded, discussed, consulted, and resolved with a senior author (VER) if necessary. Inter-rater agreement will be measured via a kappa statistic.

### Data collection

We will adapt a Data Extraction Template for Cochrane Reviews [79] to extract information from the selected studies. Both reviewers will independently extract the data from selected studies, and the information will be cross-checked, while any discrepancies will be resolved through discussion between reviewers and a senior author. If necessary, corresponding authors of the eligible studies will be contacted. The abstracted data will be collected and organized in the MS Excel spreadsheet.

### Data items

Information related to the patient population, study characteristics, and interventions will be extracted from each included study. Patient characteristics will include but not be limited to age, with OHCA; length of hospitalization; and information on possible confounders, needed for inclusion in final data analysis, such as gender, co-morbid conditions such as diabetes, polypharmacy, DNR status, access to post-resuscitation interventions, e.g., rural vs. urban environment or lack of cardiovascular unit/PCI center in the hospital, year of the incident in order to account for introduction of the post-arrest care guideline recommendations. In addition, outcome data pertaining to the utilization of relevant interventions will be collected: coronary angiography +/–, percutaneous coronary intervention, therapeutic hypothermia, implantable defibrillators, coronary artery bypass graft, and neuroprognostication ≥ 72 h. Study characteristics will record but not be limited to study design, intervention and comparators, sample size, year of study conduct, year of publication, and country of origin.

### Risk of bias and quality assessment of individual studies

In order to assess the quality of the studies included in this systematic review, a team of three reviewers (JB, JW, and VER) will use the modified Downs and Black scale (Table 2). The Downs and Black assessment tool [80] was selected for the following reasons: (i) in an evaluation by Deeks et al., it was one of the six instruments considered most suitable for use in systematic reviews of non-randomized studies, out of 194 tools identified [81]; (ii) it was recommended as one of the two most useful tools for assessing risk of bias in non-randomized studies by Cochrane Collaboration [77]; and (iii) in an appraisal by the Agency for Healthcare Research and Quality (AHRQ), it was the only quality rating system for observational studies that addressed eight of the nine domains and had a high inter-rater reliability [82].

**Table 2:** Modified Downs and Black quality scoring system

Quality indicators	Points
Reporting	
Is hypothesis/objectives/aim clearly described (1)	
Are the main outcomes to be measured clearly described in the introduction/methods (1)	
Are the characteristics of the study/population clearly described (1)	
Is the distribution of confounders among study groups clearly described and did the study adjust for those confounders (1)	
Are the main findings described (1)	
Have characteristics of patients lost to follow-up/did not complete the study been described (1)	
External validity	
Are study participants representative of entire population from which they were recruited (1)	
Are study participants representative of current OHCA population (1)	
Were those participants who were prepared to participate representative of the entire population from which they were recruited (1)	
Internal validity	
Was an attempt made to blind those measuring main outcomes of the study (1)	
Did the study adjust for different lengths of follow-up of participants (1)	
Were statistical tests used appropriate (1)	
Was the outcome measure accurate (1)	
Were the participants in the different groups recruited from the same populations (1)	
Were study participants in the different groups recruited over the same time period (1)	
Was there adequate adjustment of confounding in the analyses (1)	
Study design	
Cross sectional (0.5)	
Cohort prospective (2)	
Cohort retrospective (1)	
Control group (yes = 1 point, no = 0 points)	
Secondary/retrospective analysis (1)	
Sample size adequate/power analysis (yes = 1, no = 0)	
Total max points for study design (4 points)	
Total points (20)	

The Downs and Black scoring tool was a checklist created to examine the methodological quality of both randomized and non-randomized studies of health care interventions. It reported an internal consistency (KR-20) of 0.89, a high test-retest reliability (*r* = 0.88) and a high inter-rater reliability (*r* = 0.75). As some items of the scale were only applicable to randomized controlled trial and no item addressed baseline comparability, the modified Downs and Black scale from Samra et al., [83] will be adapted for our purpose. An additional item of “Cohort study retrospective” worthy of one point will be added to the study design category.

### Synthesis of results

The primary focus of this study is to determine the association between age and the likelihood of receiving a specific intervention as part of the post-arrest care. Age will be mainly treated as dichotomous variable (using a 65-year threshold) in accordance with the clinical definition of elderly. However, depending on the availability of data, age will also be considered a continuous variable, in order to potentially identify non-linear relationships between age and intervention reception.

Statistical methods for evidence synthesis, such as meta-analysis and meta-regression, will be applied to compare and combine the evidence regarding the association between age and intervention provision/utilization, adjusting for a number of significant confounders, such as patient characteristics and co-morbidities and availability of intervention techniques, as well as study specific characteristics.

Emphasis will be placed on variables regarding the frailty of the participants. Although it is expected to be correlated with older age, frailty encompasses a large number of combined characteristics, symptoms, and illnesses that neither preclude nor always come with older age. Since it is clinically justifiable to hesitate administrating some of the interventions of interest to frail patients, it would be important to adjust for the level of frailty when investigating the association between age and provision/utilization. Therefore, information regarding frailty levels or surrogate measures will need to be extracted from the studies in order to be accounted for in the analysis.

Since it is anticipated that not all of this information will be available from all participating studies, the selected statistical methods need to accommodate missing data as well as heterogeneous sources of data. Bayesian methods and graphical models are examples of these types of methods [84].

### Strength of the evidence assessment

The strength of evidence from the selected studies will be assessed using a modified Grades of Recommendation, Assessment, Development, and Evaluation (GRADE) system [85]. The GRADE approach is recommended by the Cochrane Collaboration as the method of choice for the evaluation of the body of evidence in systematic reviews [77]. We will assess the quality of evidence across several domains, such as risk of bias, consistency, directness, precision, and publication bias. In addition, systematic risk of over- or under-estimate of effect due to selective publication of studies using funnel plots will be utilized.

## Discussion

The goal of this review is to determine if discrepancy in the provision of post-arrest care exists for the elderly population, based on the existing published literature. The plan is to analyze data extracted from studies on OHCA patients who survived to hospital admission. Study inclusion criteria would include but not be limited to a selected number of key evidence-based interventions and age stratification.

In conclusion, this review will establish the existing link between long-term survivorship and use of evidence-based interventions in the OHCA elderly population. The review will also point out the possible gap in the literature on that issue. Lastly, the aim of this protocol and the future systematic review is to contribute by making a full search strategy, methods, and assessments accessible to those interested in studying marginalized populations such as elderly.

## Additional file

Additional file 1: The PRISMA-P checklist has been utilized and uploaded as required by the Systematic Reviews author guidelines. (DOC 80 kb)

## Abbreviations

AHRQ: Agency for Healthcare Research and Quality
CAGB: coronary artery bypass graft
CARES: Cardiac Arrest Registry to Enhanced Survival (US)
DNR: do-not-resuscitate
EMS: emergency medical services
GRADE: Grades of Recommendation, Assessment, Development, and Evaluation
ICD: implantable cardioverter-defibrillator
MeSH: Medical Subject Headings
OHCA: out-of-hospital cardiac arrest
PCI: percutaneous coronary intervention
PRESS: Peer Review of Electronic Search Strategies
PRISMA: Preferred Reporting Items for Systematic Reviews and Meta-analyses
PRISMA-P: Preferred Reporting Items for Systematic Review and Meta-analysis Protocols
RCT: randomized controlled studies
ROSC: return of spontaneous circulation
SR: systematic review
